# Using machine learning to understand the implications of meteorological conditions for fish kills

**DOI:** 10.1038/s41598-020-73922-3

**Published:** 2020-10-12

**Authors:** You-Jia Chen, Emily Nicholson, Su-Ting Cheng

**Affiliations:** 1 School of Forestry and Resource Conservation, National Taiwan University, No. 1, Sec. 4, Roosevelt Rd., Taipei, 10617 Taiwan, ROC; 2 Centre for Integrative Ecology, School of Life and Environmental Sciences, Deakin University, Burwood Campus, Melbourne, Australia

**Keywords:** Climate sciences, Ecology, Environmental sciences

## Abstract

Fish kills, often caused by low levels of dissolved oxygen (DO), involve with complex interactions and dynamics in the environment. In many places the precise cause of massive fish kills remains uncertain due to a lack of continuous water quality monitoring. In this study, we tested if meteorological conditions could act as a proxy for low levels of DO by relating readily available meteorological data to fish kills of grey mullet (*Mugil cephalus*) using a machine learning technique, the self-organizing map (SOM). Driven by different meteorological patterns, fish kills were classified into summer and non-summer types by the SOM. Summer fish kills were associated with extended periods of lower air pressure and higher temperature, and concentrated storm events 2–3 days before the fish kills. In contrast, non-summer fish kills followed a combination of relatively low air pressure, continuous lower wind speed, and successive storm events 5 days before the fish kills. Our findings suggest that abnormal meteorological conditions can serve as warning signals for managers to avoid fish kills by taking preventative actions. While not replacing water monitoring programs, meteorological data can support fishery management to safeguard the health of the riverine ecosystems.

## Introduction

Massive mortality of fish, known as fish kill, is a common phenomenon around the world^1–5^. Negative impacts of fish kill on river ecosystems include declines in fish populations, degradation of water quality^3,4^, and socio-economic costs involved in cleaning up dead fish that affect amenity values^6^. While fish kills can be attributed to a wide range of reasons, such as eutrophication, high ammonia concentration, heat exhaustion and disease^3,7,8^, a common cause is the level of dissolved oxygen^8,9^. Exhaustion of localized DO, acute reductions of DO to hypoxia (i.e., DO < 2 mg/L)^10,11^, and/or any kind of DO depletion pose real threats to fish that can lead to low-dissolved oxygen syndrome and death^3,9,10^. For example, in the Mary River in Australia, fish kill events occurred when river flow carried oxygen-consuming materials that depleted DO during the wet season^12^.

The DO concentration is determined by rates of oxygen supply and consumption, so that processes of air–water exchange, photosynthesis, respiration, organic matter decomposition, nitrification, sediment oxygen consumption could directly or indirectly interact or combine to influence the DO concentration and, in turn, cause fish kills^10,13,14^. While DO is responsible for many fish kill events, intermittent monitoring of water quality (e.g., monthly or less frequently), means that direct causation can be difficult to attribute, and periods of high risk of fish kill cannot be detected in time to implement preventative measures. The lack of water quality monitoring data poses real challenge for riverine ecosystem management for many places worldwide.

Given that the diffusion of oxygen between air and water interface is a two-way reaction, meteorological measurements could provide some understanding of conditions that may impede the dissolubility of oxygen into the water, leading potentially to fish kill events. Interactions between meteorological factors on the amount of oxygen dissolved in water are complex^15,16^: temperature controls the saturation concentration of DO^17–19^; precipitation washes oxygen-consuming material into rivers^11,12^; and wind speed promotes DO through air–water oxygen diffusion by creating rough surfaces^20,21^. Other weather-related conditions can also indirectly affect DO, such as photosynthesis-related factors like temperature, nutrients and solar radiation, and respiration-related factors such as organic matter decomposition by microbes, carbonaceous biochemical oxygen demand (CBOD) and total organic carbon (TOC)^17,22^. Although these complex interactions present challenges in relating meteorological mechanisms to levels of DO in water, continuous meteorological observations are typically taken in many places around the world. Depending on the extent to which the meteorological factors can explain in relation to the DO conditions, any nonlinear relationship behind the fish kills might be revealed by new analytical approaches.

In this study, we apply a machine learning technique to test if meteorological measurements could act as a proxy for low levels of DO in the absence of continuous water quality monitoring data, to predict massive fish kills and provide management guidance. We used fish kill events of the grey mullet (*Mugil cephalus*), in the lower Danshui River in Taiwan as a case study. This species presents an ideal case study because multiple fish kills have been reported throughout the year and attributed to a sudden drop of dissolved oxygen (DO). Yet DO is monitored only once a month, or measured after the fish kill events occurred, making the detection or prevention of fish kills difficult. The species also has dynamic spatial and temporal interactions with its environment, including localized diel movements and seasonal or life-cycle movements with the ambient physical environmental conditions along their upstream or downstream passage^23,24^, covering habitats of seawater, brackish water and freshwater environments, and estuaries in a relatively short period of time^4^. Our specific objectives include: (1) gathering historical fish kill events in the lower Danshuei River (Taiwan) as a case study and the associated hourly meteorological measurements close to these events; (2) applying the self-organizing maps (SOM) as a non-biased clustering tool to explore the nonlinear relationships between the various meteorological factors and fish kills; and (3) identifying early warning signals behind the meteorological patterns associated with fish kills to implement timely actions for mitigation.

## Results

Based on the clustering results of the SOM, followed by a systematic risk analysis, our results suggested that the occurrence of fish kills can be categorized into different types, and different meteorological stressors can cause cumulative effects that increase the risk of fish kills.

### Recognize types of fish kills by the SOM

**Figure 1:**
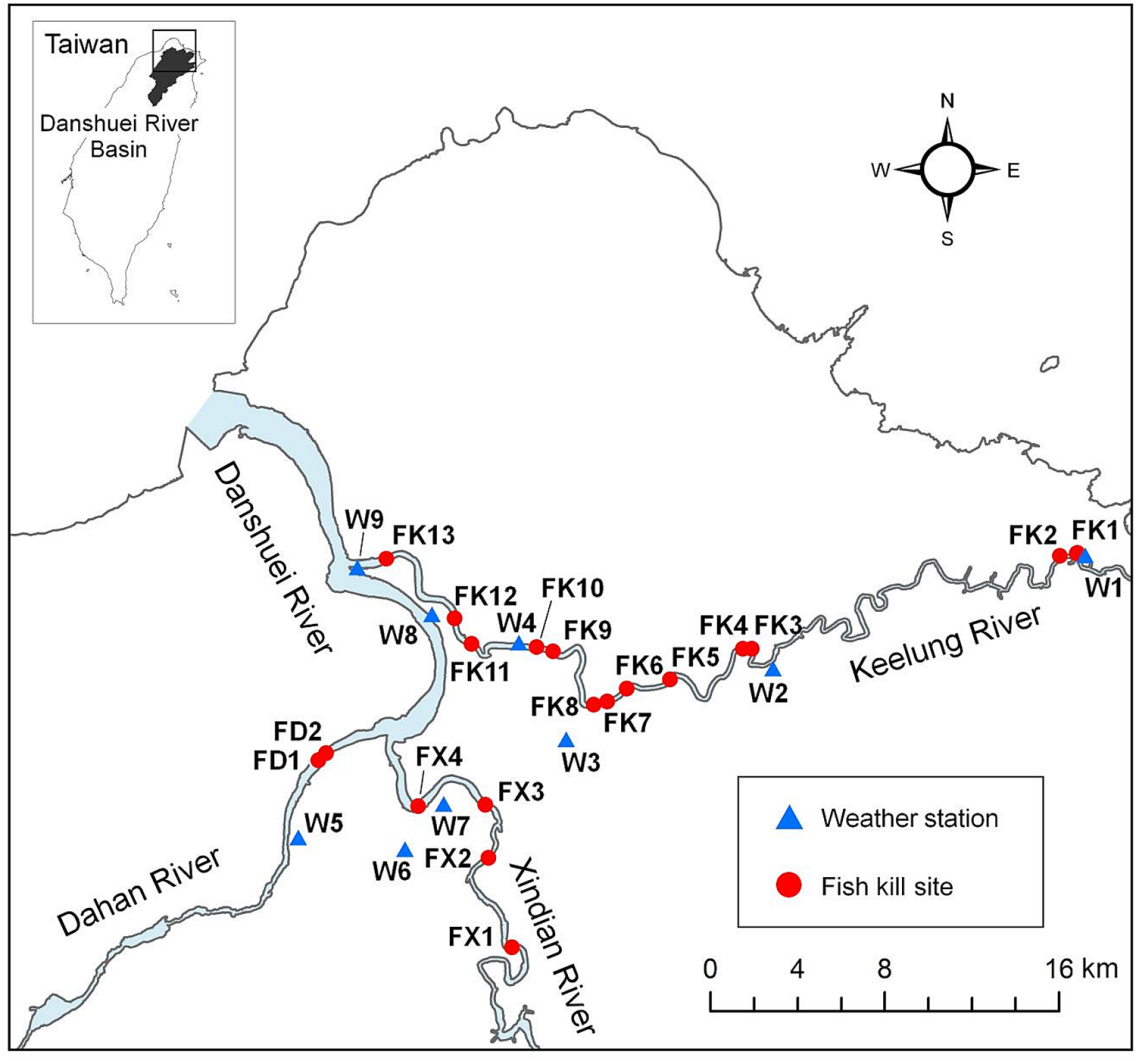
The fish kills occurred in the downstream of each tributary of the Danshuei River (red dots in the figure), and we gathered meteorological data from the nearby weather stations (blue triangles in the figure). This map was generated using ArcGIS version 10.5.

From year 2010 to 2018, more than 27 grey mullet fish kill events were recorded in the lower Danshuei River. Excluding those clearly resulted from industrial pollution and those missing required meteorological observations, the final 19 events analyzed occurred across 19 sites in the months of April to September (Fig. 1) with estimated weights of dead fish ranging from 1500 to 35,000 kg (Table 1).

**Table 1:** Detailed information of the assembled 19 fish kill events in one of the three tributaries (i.e., Dahan, Keelung, or Xindian River) of Danshuei River.

Event ID	Tributary name	Reported date of fish kill event	Estimated weight (kg)	Reported fish kill site	Nearby weather station	Reporting media
1	Dahan	2016/4/8	NA	FD1	W5 (Banqiao)	Liberty Times Net
2		2018/8/13	35,000	FD2	W5 (Banqiao)	United Daily News
3	Keelung	2010/5/10	2000	FK7	W3 (Xinyi)	United Daily News
4		2011/4/18	3000	FK9	W4 (Dazhi)	Liberty Times Net
5		2011/10/13	3000	FK10	W4 (Dazhi)	Apple Daily
6		2013/6/23	NA	FK8	W3 (Xinyi)	China Times
7		2013/7/25	7000	FK6	W3 (Xinyi)	Liberty Times Net
8		2014/9/11	NA	FK5	W2 (Xizhi)	Taipei City Government Environmental Protection Bureau
9		2015/7/20	1,500	FK11	W4 (Dazhi)	Liberty Times Net
10		2016/4/26	NA	FK1	W1 (Rueifang)	Liberty Times Net
11		2016/5/27	NA	FK2	W1 (Rueifang)	Keelung City Garbage collection site
12		2016/7/21	NA	FK13	W9 (Shezih)	Apple Daily
13		2017/7/29	The Liberty Times Net estimated a total weight of 194,190 kg for events occurred during late July to August in 2017	FK3	W2 (Xizhi)	United Daily News
14		2017/8/12		FK4	W2 (Xizhi)	New Taipei City Government Environmental Protection Bureau
15		2017/8/28		FK12	W8 (Shihlin)	Taipei City Government Environmental Protection Bureau
16	Xindian	2011/4/18	3000	FX2	W7 (Yonghe)	Liberty Times Net
17		2012/4/19	> 2000	FX4	W7 (Yonghe)	Taiwan Environmental Information Association
18		2014/9/11	27,700	FX3	W7 (Yonghe)	Taipei City Government Environmental Protection Bureau
19		2017/8/10	NA	FX1	W6 (Zhonghe)	Liberty Times Net

**Figure 2:**
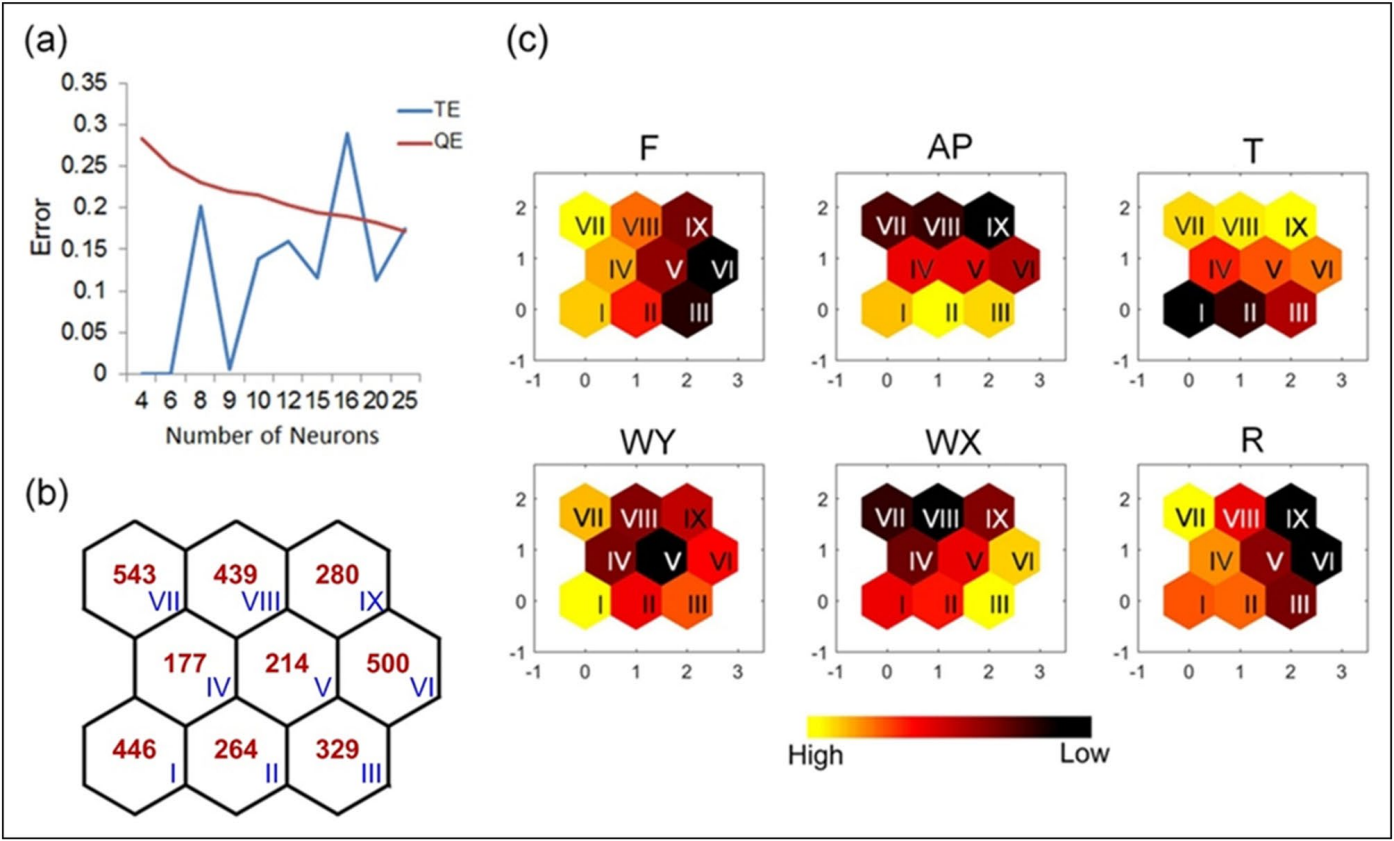
(**a**) Map size was determined to be 9 neurons (i.e., 3×3) based on the measurement of quantization error (QE) and topographic error (TE); (**b**) number of input records (shown in red) in each neuron (shown in blue); (**c**) results of the SOM representing associations of meteorological conditions of air pressure (AP), temperature (T), wind speed in N–S direction (WY) and in E–W direction (WX), and precipitation (R), to the reported time of fish kills (F).

By bundling each fish kill event with associated hourly weather data into a parallel input matrix form from 7 days before each event, we assembled a total of 3192 data matrix (i.e., 19 events × 7 days × 24 hours = 3192) for 6 variables of reporting time of fish kills (F), air pressure (AP), temperature (T), wind speed in north–south direction (WY) and in east–west direction (WX), and precipitation (R). The criteria of local minimum of quantization error (QE) and topographic error (TE) determined a proper SOM size for interpretation to be 9 groups (i.e., a 3×3 SOM) (Fig. 2a), and neuron I and neuron VII had been assigned the most data, of which 446 and 543, respectively (Fig. 2b). In the SOM, the same neuron numbers demonstrating parallel conditions in each variable may show different colors in a band from dark brown to yellow, representing data values from small to large. These color patterns and their actual values can help relate the conditions of various variables and their non-linear inter-relationships.

Because the time the fish kills actually happened remains unknown, the value of F provided a way to estimate the temporal distance between the analyzed day and the reporting of the fish kill event. In the results, neurons I and VII were yellow-hued in the variable of fish kill time (F), representing a closer time between the meteorological conditions and the reported day of each fish kill event (day 7) (Fig. 2c). Traced back to the bundled date of each dataset in neurons I and VII, we found distinct time frames of summer fish kill type (neuron VII) and non-summer one (neuron I), where the SOM classified events 1, 3, 4, 5, 10, 11, 16, and 17 as non-summer fish kill type, and events 2, 6, 7, 8, 9, 12, 13, 14, 15, 18, and 19 as summer fish kills (Table 1).

### Seasonal meteorological variations

**Figure 3:**
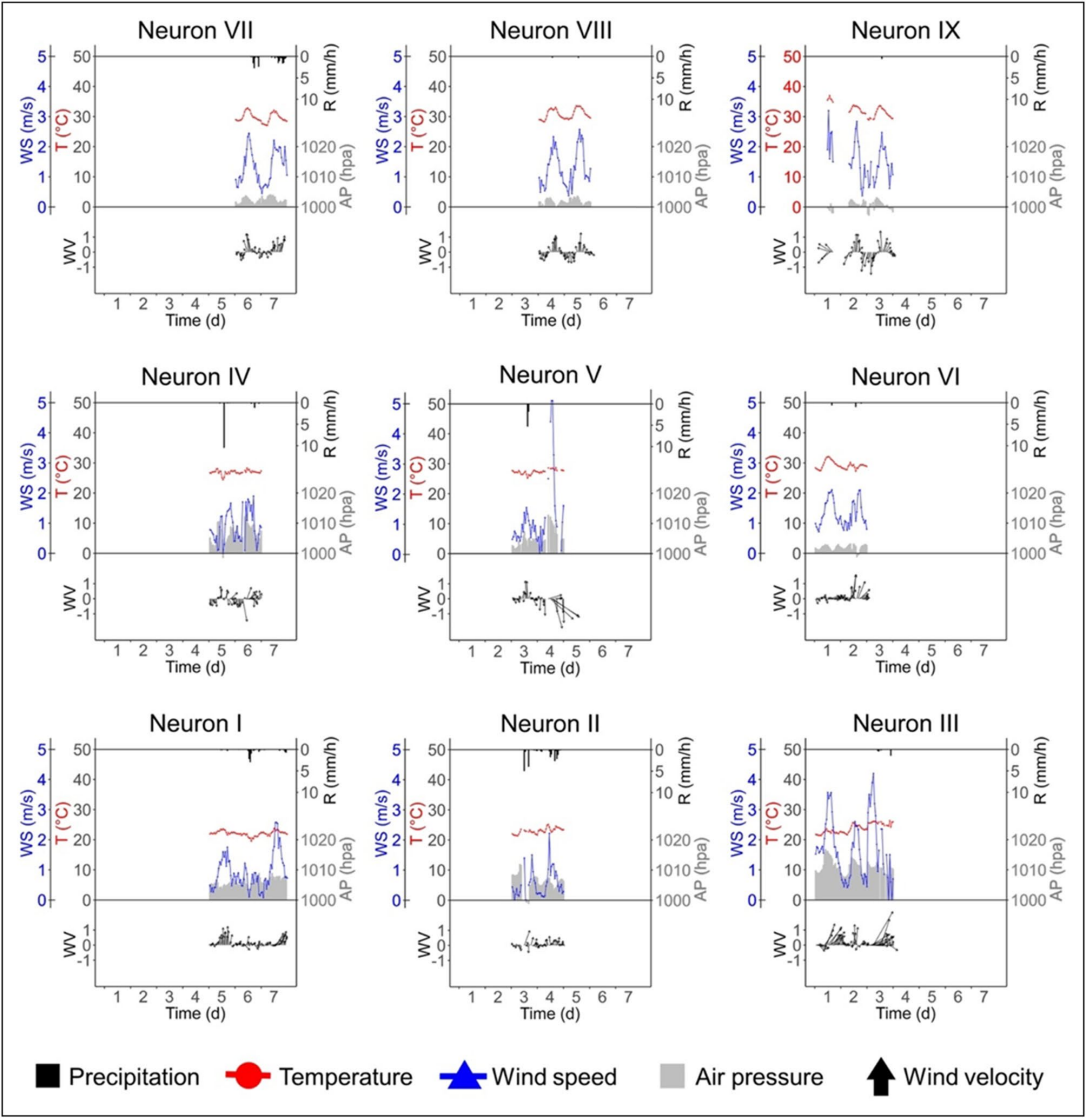
A general view of the actual meteorological conditions of AP (grey bar), T (red line), R (upper black bar), WS (blue line), and WV (bottom black arrow lines) clustered in each neuron.

Because our analysis bundled the corresponding meteorological conditions with the time of fish kill events, the parallel linked characteristics of each variable in the SOMs helped provide unbiased recognitions of the temporal non-linear and complex relationships and patterns across the heterogeneous data inputs (Fig. 2c). With the nested algorithm of SOM, the neighborhood neurons exhibited closer patterns and relationships, which facilitated an understanding of the meteorological trends associated with different fish kill types (Fig. 3). Based on the results, the summer type fish kills can be traced back to neurons IX, VIII, and VII, having air pressure (AP) gradients from 1001.1 to 1002.4 hpa and temperature (T) gradients from 32.3 to 29.9 °C across the 7 days; a sudden drop of wind in the vertical position (WY) from 0.24 to 0.09 m/s, with a bounce back to 0.24 m/s; an increasing trend in wind in the horizontal position (WX) from −0.03 to 0.24 m/s; an intense storm from the 5th to 7th day (R) at an hourly rate of 0.35 mm/h that accumulated to a total of 192.5 mm (Fig. 3 and Table 2). In contrast, the non-summer type fish kills were grouped into neurons III, II, and I with greater AP gradients from 1010.3 to 1006.1 hpa; smaller T gradients from 23.1 to 22.1 °C; a sudden drop of WY from 0.26 to 0.12 m/s with a bounce back to 0.24 m/s, and of WX from 0.80 to 0.14 m/s with a bounce back to 0.41 m/s; a longer period of continuously rain (R) since the 3rd to the 7th day at hourly rate of 0.34 and 0.13 mm/h that accumulated to a total of 90.0 and 57.5 mm in neuron II and neuron I, respectively (Fig. 3 and Table 2). Similar meteorological patterns were clustered in neurons IV, V, and VI, in which they shared patterns and gradients with the two fish kill types.

**Table 2:** Detailed information for each variable in neurons of the SOM.

Neuron number	Number of Data	AP (hpa)	T (°C)	WY (m/s)	WX (m/s)	WS (m/s)	Average R (mm/h)	Total R (mm)	Duration of R (h)
I	446	1006.1	22.1	0.24	0.41	0.83	0.13	57.5	38
II	264	1006.3	23.1	0.12	0.14	0.48	0.34	90.0	34
III	329	1010.3	23.1	0.26	0.80	1.44	0.02	5.5	6
IV	177	1005.6	26.9	−0.10	0.21	0.80	0.24	43.0	10
V	214	1003.8	27.2	−0.06	0.27	0.84	0.18	39.5	6
VI	500	1001.9	29.4	0.11	0.67	1.31	0.03	16.0	7
VII	543	1002.4	29.9	0.24	0.20	1.42	0.35	192.5	38
VIII	439	1001.9	31.2	0.09	0.04	1.42	0.02	9.5	2
IX	280	1001.1	32.3	0.24	−0.03	1.64	0.03	7.0	2
Total	3192	1004.1	27.5	0.15	0.32	1.19	0.14	460.5	–

Notably, wind speed (i.e., WS, considering WX and WY together) in the summer fish kills appeared to have a diurnal wind direction pattern (WV) and a cycled descending and bouncing back since the 2nd to the 6th days. On contrast, non-summer fish kills were associated with a constant northeastward direction with little change in wind direction, and a decreasing trend of WS that periodically dropped to almost zero m/s between the 3rd to the 6th days (Fig. 3).

### Systematic meteorological risk assessments

**Figure 4:**
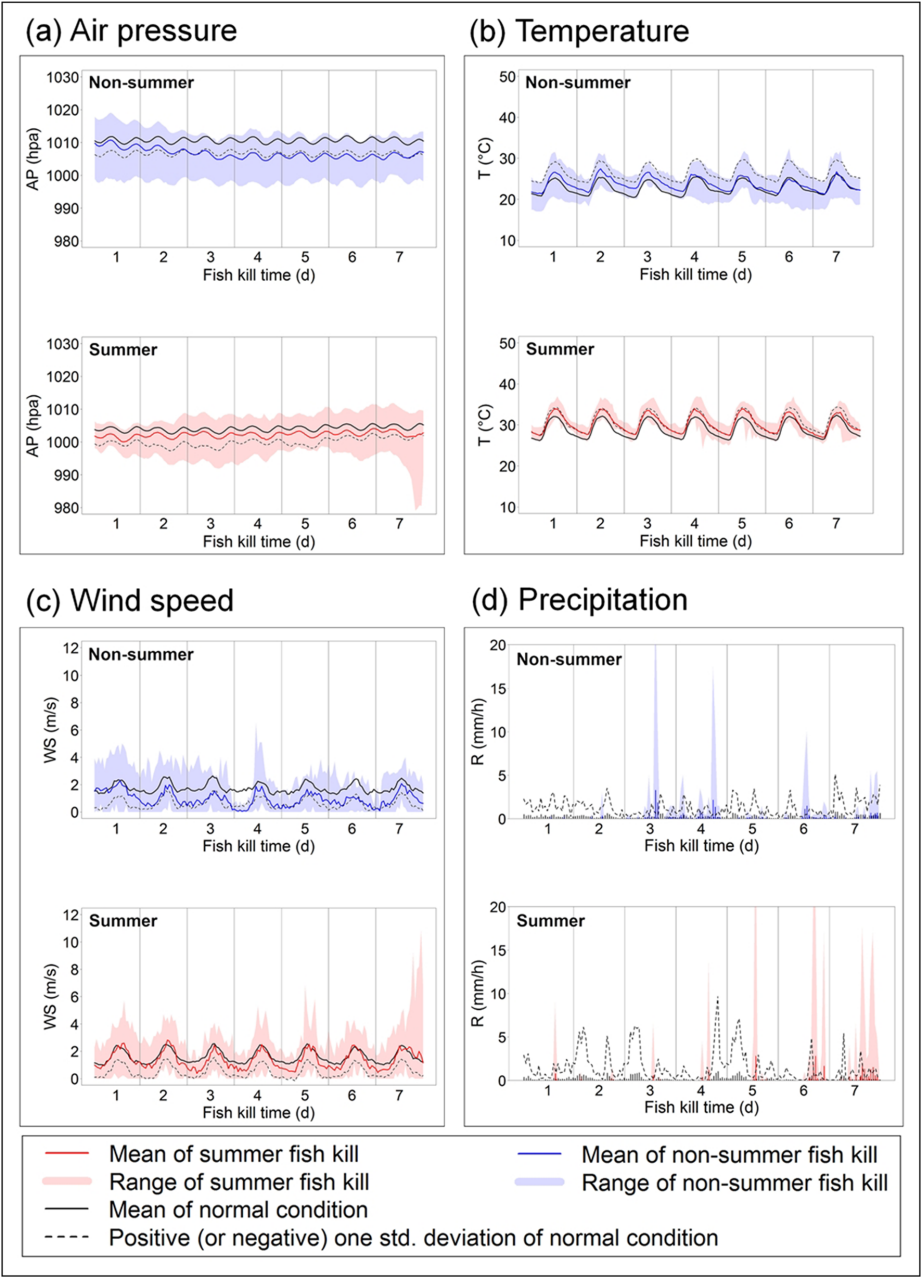
Risk assessments of the meteorological factors of (**a**) air pressure (AP), (**b**) temperature (T), (**c**) wind speed (WS), and (**d**) precipitation (R) in 2010–2018.

The risk analysis results showed distinct forcing factors to summer vs. non-summer fish kill types (Fig. 4). Comparing the normal average conditions (i.e., average of the conditions with no fish kills, black line) of multiple meterological variables of air pressure (AP), temperature (T), wind speed (WS), and precipitation (R), to those average conditions in the type of non-summer (blue line) and those in the summer fish kill type (red line), we found that in the summer fish kill type, normal AP during the summer time (black line in Fig. 4a) was about 5–7 hpa lower than that during the non-summer time, yet AP of the summer fish kills (red line in Fig. 4a) was even lower; T (red line in Fig. 4b) across the 7 days were very close to the dash line (i.e., one standard deviation around the mean), representing a boundary of a 66.7% probability to the normal conditions, compounding by periodically lower WS < 1 m/s (Fig. 4c) and concentrated storms (R) (particularly over 1.5 to 2 mm/h) in the 5th to 7th days (Fig. 4d). In contrast, meteorological tension to air–water oxygen diffusion in the non-summer fish kill type was intensified by AP (blue line in Fig. 4a) lower than one standard deviation to the normal conditions (dash line) since the 2nd to the 3rd days; higher T (blue line in Fig. 4b) in the 1st to 3rd days; much lower WS almost across the 7 days (Fig. 4c); intensive storms (R) over 1.5 mm/h in the 3rd to 4th days and the 6th days (Fig. 4d). Based on the results, the two fish kill types appear to have different critical climatic actors as barriers for oxygen difussion in the air–water interface.

**Figure 5:**
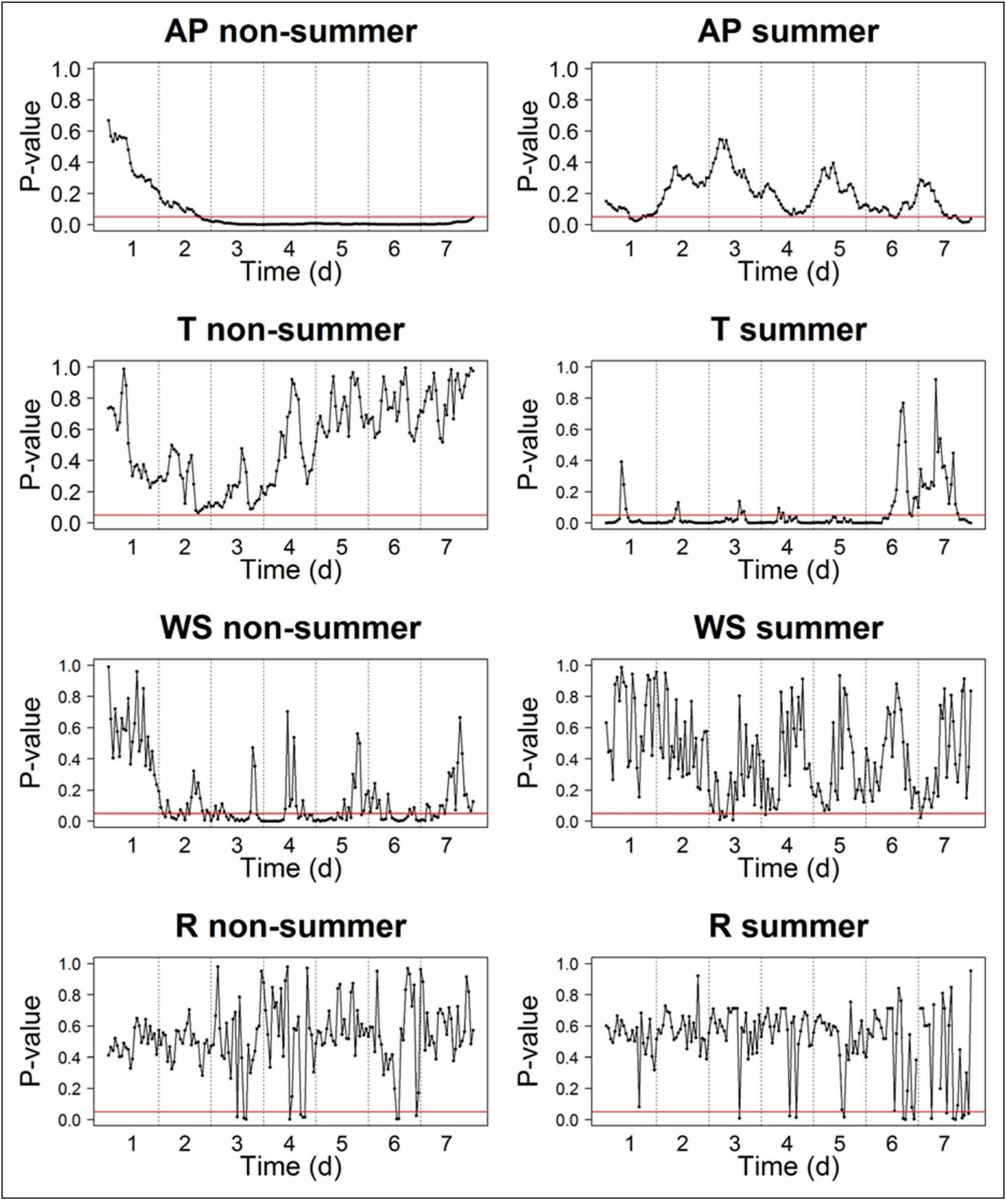
Significance level of hourly comparisons between fish kill and non-kill normal average conditions with a red line showing the significance level of 0.05.

In addition, we tested the hourly differences between fish kill and non-kill average conditions. We found that temperature (T) had a significance level lower than 0.05 for most of the time in the summer fish kill type, while air pressure (AP) and wind speed (WS) more frequently had a significance level lower than 0.05 for the non-summer fish kill type (Fig. 5). Other variables were largely above the 0.05 level of significance (Fig. 5). This suggested that summer fish kills were mostly induced by T, and intensified by AP and precipitation (R); while non-summer fish kills appeared to be triggered by AP and WS, and worsened by R. As a result, if the average meteorological conditions of AP, T, R, and WS without fish kills is assumed to be normal states of non-kill conditions, our study indicates threshold changes that may serve as early warning signals for practical monitoring or fish kill prevention purposes: purturbations over the range of one standard deviation of T and R, and negative one standard deviation of AP and WS (Table 3). Given the cumulative adverse impacts of each meteorological factor (Fig. 5), when any of the meteorological conditions hit the above mentioned threholds, it can be seen as a warning signal; with two or more meteorological conditions satisfied the warning thresholds, preventative actions are recommended.

**Table 3:** Summary of daily normal average conditions and values of positive or negative one standard deviation for each meteorological variable during non-kill summer and non-summer periods.

**Summer**								
Variable		Day 1	Day 2	Day 3	Day 4	Day 5	Day 6	Day 7
AP (hpa)	Min of normal ave.	1003.8	1003.7	1003.6	1004.0	1004.2	1004.7	1004.7
	Min of −1 std. dev.	998.8	997.3	997.4	999.2	998.1	1000.5	1000.5
T (°C)	Max of normal ave.	28.9	29.0	28.9	28.9	28.8	28.9	29.0
	Max of +1 std. dev.	34.3	34.0	34.1	34.2	34.3	34.3	34.4
WS (m/s)	Min of normal ave.	1.56	1.62	1.65	1.59	1.62	1.55	1.57
	Min of −1 std. dev.	0.07	0.00	0.00	0.03	0.00	0.05	0.08
R (mm/h)	Max of normal ave.	0.2	0.3	0.3	0.2	0.3	0.1	0.1
	Max of +1 std. dev.	3.1	6.1	6.3	9.7	7.2	4.9	5.4

**Non-summer**								
Variable		Day 1	Day 2	Day 3	Day 4	Day 5	Day 6	Day 7
AP (hpa)	Min of normal ave.	1010.7	1010.7	1010.8	1010.6	1010.5	1010.9	1010.5
	Min of −1 std. dev.	1005.3	1005.6	1005.9	1005.5	1005.4	1005.6	1005.1
T (°C)	Max of normal ave.	22.7	22.7	22.4	22.9	23.0	22.9	23.3
	Max of +1 std. dev.	29.2	29.5	29.1	29.9	29.7	29.6	29.6
WS (m/s)	Min of normal ave.	1.76	1.80	1.85	1.69	1.72	1.69	1.76
	Min of −1 std. dev.	0.10	0.18	0.23	0.20	0.13	0.10	0.06
R (mm/h)	Max of normal ave.	0.3	0.3	0.2	0.2	0.2	0.2	0.4
	Max of +1 std. dev.	3.1	3.5	2.9	2.8	3.4	3.3	5.1

## Discussion

Due to the lack of continuous water quality monitoring data, the causal effects of ambient environmental conditions on fish kills has been difficult to predict in many places. In this study, we showed how machine learning techniques can be applied for the classification of spatial and temporal information associated with the meteorological conditions to discriminate patterns in fish kill events. We took historical fish kill events of the lower Danshuei River as a case study to explore the potential influences from the explicit meteorological conditions on fish kills in the circumstances of intermittent DO monitoring data and seek for implications on future fish kill mitigations. We found that without continuous water monitoring data, available meteorological observations can provide a useful early warning to allow timely action for fish kill avoidance.

Fish kills were grouped by the SOM into time-dependent “summer” versus “non-summer” fish kill types. Although the summer/non-summer classification seemed not a difficult classification, the non-linear relationships across a range of multiple factors with various response time and effects are hard to detect by conventional techniques. This study enabled a way to visualize complex interactions from the meteorological conditions conducive to the occurrence of fish kills with a consideration of time dependent variations in a 7-day time series. Summer-type fish kills are associated with low air pressure, high temperatures and a prior 2–3 days concentrated precipitation; in contrast, non-summer events resulted from compounding effects of low pressure, low wind speed, and longer periods of intensive storms to cause death of fish. These results provide a science-based foundation to disentangle the mystery and clarify the potential conditions causing fish kills.

In general, it has been reported in the news that the main problem causing fish kills is the high temperature resulting in a sudden hypoxia condition of the water leading to massive death of fish. Indeed, in our analysis high temperature was a critical factor to form summer type fish kills (Fig. 4b). Yet focusing on temperature alone cannot fully account for the complex causes of the fish kills. Our results revealed that the lethal circumstances in both the summer and non-summer fish kill types were compounded by and attributed to concentrated storms that were surmised to stir up bottom sediments because of the higher water level and flow rate contributed from the storms. This effect was also found in the fish kills in Australia, highlighting the significant impact from stormwater runoff transporting a substantial organic load with high oxygen demand^25^. Floodplain and estuarine water bodies, both as a part of the riverine system, often receive large amount of sediments carrying excessive anthropogenic inputs of nutrients and organic matter^26^ that can easily exhaust the remnant oxygen in the water. Consequent death associated with serious hypoxia situation could happen across large areas without leaving any refugee or sheltering places^27^. Lower air pressure and prolonged diurnal pattern of wind direction with low wind speed may have additional harmful effects that reinforces the circumstance by breaking down the required oxygen diffusion mechanism from the atmosphere to the water^28^, eventually leading to a lethal outcome.

Our results suggested that fish are vulnerable to combinations of driving forces, which, when they exceed the survival requirements, lead to fish kills. This is why in a situation of lower temperature and higher air pressure that theoretically should facilitate the diffusion of oxygen^19,21^, fish kills still occur. Nonetheless, fundamental knowledge to the understanding of oxygen exchange dynamics among the atmosphere, fresh water, tide, and sediments, and the resultant stresses affecting the fish is still incomplete. We can only confirm that the occurrence of these fish kills implies greater mixed disturbances preventing effective oxygen diffusion into the water, such as a decreasing trend of air pressure^21^, a drop of wind speed to a low magnitude, a constant wind direction without obvious change throughout the week, and a successive precipitation^11^, and that these multiple players’ status along the temporal horizon could together proceed to structure a dramatic barrier leading the river water into a hypoxia situation.

The results showed distinct combination of meteorological stressors for the two types fish kills; this understanding of the potential forcing stressors from available meteorological data could provide practical information for fish kill preventive actions for places lacking essential dissolved oxygen monitoring. When abnormal meteorological conditions hit the threshold of possible fish kill occurrence, they can serve as warning signals for managers to take preventative actions, such as proactive water oxygenation, to avoid fish kill. This is particularly important under climate change. For example, in Taiwan the diurnal and annual temperature changes had been increased by 1 to 1.4 °C in the past 100 years^29^, as well as the uneven trend of precipitation in space and time^30^, in which more intense storms and about 80% annual precipitation concentrated are projected to occur in the wet season in the middle, southern and eastern parts of Taiwan, while less rain in the dry season with warnings on more successive droughts^31^. This may imply greater fluctuations in air temperature and more washed off terrestrial nutrients by larger precipitation intensity, as well as bottom-sediment disturbance associated with changes in the river flow affected by floods or droughts^30^.

A serious problem can arise due to IPCC’s forecasting on longer hot days under global warming^32,33^. Under high temperatures, there might appear an increase in the oxygen demand of aquatic animals^34^ and sediment oxygen demand. These negative situations will recurrently form DO anomalies, and even be intensified by more urbanization-caused anthropogenic organic matters being brought into the estuaries^35^, potentially leading to more frequent and larger areas of fish kills^36^. During the summer time, DO conditions could be harsh due to higher temperature and lower air pressure. Several fish species are known to survive under very low DO concentrations because under progressive hypoxia, the adult fish are forced to depress aerobic and enacted anaerobic metabolism to extend their survival^37^. Nonetheless, larger body-size adults tend to have higher oxygen demand, and therefore, when expose to acute hypoxia, larger body-size adults may be more sensitive to oxygen deficits^38^.

Considering the life-cycle movements of grey mullet with the changes of their inhabitant environmental conditions, the dominance observed in the non-summer type fish kills were the juveniles and the younger grey mullets^39^, because they start to spawn in the estuary in late fall to winter time; eggs hatch and the juveniles utilize the estuary as a nursery ground in November to March; then they migrate upstream to freshwater feeding areas. While the potential individuals dead in the summer type fish kills were the larger grey mullet adults, which were the matured adults migrating downstream to go back to the sea during the summer time^39^. Since the two types of fish kills targeted on individuals in different life stages, the population structure of grey mullets may be impacted; this will require further study to confirm. Such analysis will rely on many years of monitoring to determine the link between physiology and life history^40^, natural fluctuation in population size, and the potential distribution of grey mullets and changes attached to it, to reflect long-term and short-term changes at the population level process for fishery management and conservation.

We suggest that long-term monitoring for finding solutions to emergent problems like fish kills requires a systematic consideration to detect where, how, and to what extent the environmental changes would become stressors to the ecosystem. Although in this study we gained insights by applying SOMs to examine the linkages of fish kills among the meteorological factors, better understanding of how and to what extent the environmental changes would act together to induce fish kills requires an extensive monitoring program of freshwater ecosystems. We recommend that developing a continuous long-term monitoring of flow and water quality at outbreak places is necessary for future research looking into the physical characteristics of the rivers to improve understanding and prediction of fish kills. In addition, this information could help flag potential sites for monitoring stations and inform the design of the long-term monitoring program. Moreover, as river networks are connected from upstream to downstream, occurrence of soil erosion or landslides, and existence of landuse change in the upstream can disrupt the balance of natural regimes and transport inputs of sediments and nutrients into downstream areas^35,36^. Major modifications such as reservoirs situated in the middle, excessive nutrient loading from agriculture^41^, pollution and biological invasions are also management-relevant element on a long-term continuum of change^42,43^. Such long-term monitoring program can have multiple aims covering a wide range of environmental and biological measures across spatial and temporal scales from upstream to downstream for detecting and/or identifying where, how, and to what extent exists considerable variabilities to influence the ecosystem health.

## Methods

### Case study and data description

In the Danshuei River, the third longest river in Taiwan, fish kill events have occurred periodically at downstream of each tributary (Fig. 1) almost every year for the last 20 years. The mainstem of the Danshuei River has a length of 158.7 km with approximately 2726 km^2^ of the drainage basin. There are three main tributaries originating from mountainous areas: the Xindian and the Dahan Rivers, which merge at Jiangzicui, and the Keelung River, which enters the mainstream of the Danshuei River at Guandu, eventually flowing into the Taiwan Strait^26^. Natural and undisturbed areas exist at upper watersheds but are intermixed with few small scale agricultural developments, where landuse patterns shift to larger scale agriculture and urban areas at the flatter terrain of river valleys and downstream areas^42^. To meet the human demands in drinking water and flood control, check dams, reservoirs, and levees have been constructed throughout the river basin and have greatly modified the riverine habitats^42^.

Located in the subtropical zone, the Danshuei River Basin is in general, humid and warm. Annual precipitation is abundant with no obvious dry season. In winter, precipitation stems from the northeast monsoon, while in summer, the heavy rains (May/June) and typhoons. In the Danshuei weather station during the last decade, annual average precipitation is around 2138.5 mm with various monthly patterns—the highest in June, reaching 323.5 mm in average, and the lowest in July, only 99.7 mm; annual monthly average temperature is around 22.6 °C, in which the highest (29.3 °C) occurs in July, and the lowest (15.6 °C) in January (interpreted data obtained from the Central Weather Bureau of Taiwan).

The grey mullet (*Mugil cephalus*) have been reported in the news as the main species in these fish kill events. The massive death of grey mullets is typically reported by the local media as the cause of high temperature resulting in low DO concentration. Here we sought to understand the relationship between fish kills and the meteorological conditions (air temperature, wind, air pressure, and precipitation). Therefore, we assembled reported grey mullet fish kill events from the online news from 2010 to 2018 (Fig. 1 and Table 1). These events occurred at multiple places within the catchment (Fig. 1), but rarely in the estuary. Assessments of the water and dead fish were evaluated by the Department of Environmental Protection (DEP) of the Taipei and New Taipei cities immediately (few hours to one day) after a single fish kill event to clarify if fish kills were caused by industrial contaminants. Based on the reports, we excluded those caused by industrial water pollution. We also gathered spatially explicit, hourly meteorological data collected by the Central Weather Bureau, including air pressure, temperature, wind speed, wind direction, and precipitation. Missing data were imputed through linear interpolation^44^.

### The Machine learning approach: the self-organizing map (SOM)

The self-organizing map (SOM) is a type of artificial neural networks, usually used as a tool for clustering or data-mining^45,46^. Its unsupervised character makes it useful in providing automatically and unbiased clustering results, by applying the “shortest relation distance” algorithm between every input variable to decide the weight vector through learning about the input data^46,47^. As the SOM can effectively reduce high data dimensions into a 2-dimensional map for clustering and visualizing, it has been widely used to explore problems in industry, natural sciences, ecology, and many other fields^48–50^.

During the SOM learning and training process, we inspected the consistency of the results to judge if convergence was reached. Evaluation was done by calculating the similarity of the SOM using the simple matching coefficient (SMC), in which a neighborhood matrix is created with both the number of rows and columns being equal to the number of data^51^, and each row or column is used to represent each data vector. In this neighborhood matrix, if two data points are assigned to the same neuron or the adjacent neuron in the SOM, the corresponding value in the matrix is 1, otherwise the value is 0. If the corresponding position of the two matrices is 1, it is regarded as positive similarity, whereas 0 is regarded as negative similarity. In the end, SMC is calculated by dividing number of matches (positive similarity and negative similarity) by the total number of elements in the matrix^51^:

$${\text{SMC}} = \frac{{\text{number of matches}}}{{\text{total number of elements in matrix}}}$$

To determine the optimal output neuron numbers of the SOM, we trained the SOM with different map sizes, including 2×2, 3×2, 3×3, …, 5×5, and applied the criteria of quantization error (QE)^52^ and topographic error (TE)^49^. In particular, we calculated the associated QE as the average distance between input vector and the weight vector of its best-matching unit (BMU)^49^:

$${\text{QE}} = \frac{1}{n}\mathop \sum \limits_{i = 1}^{n} \left| {\left| {x_{i} - u_{c} } \right|} \right|,$$

where x_i_ is the input vector, u_c_ is the vector of the BMU, and n is the number of data vectors. We considered the number of input vectors that its second-matching unit (SMU) is not adjacent to the BMU as the error of TE^49^:

$${\text{TE}} = \frac{1}{n}\left( {\mathop \sum \limits_{i = 1}^{n} u\left( {x_{i} } \right)} \right),$$

where u (x_i_) is set to 1 if the SMU is not adjacent to the BMU.

Moreover, since QE decreases when output neuron numbers increase, we determined the optimal solution as the local minimum of TE^49^, and took the shape of the SOM map into consideration for easier visualization purposes. As a result, the square shaped map (i.e., same neuron numbers in length and width) was preferred since it retained patterns among input variables whichever the SOM map was rotated.

### Modeling procedure

To explore the relationship among fish kills and multiple meteorological factors, we took hourly weather data of air pressure (AP), temperature (T), wind speed (WS), wind direction (WD), and precipitation (R) to compare with the “fish kill time” (F) representing the days to the reported fish kill news for analysis. Under this setting, the reported date of fish kill was set as time 7, and bundled with hourly weather data from 7 days before the event (i.e., time 1 to time 7). To present the value of a cyclic variable (i.e., the wind direction), yet to preserve the magnitude of wind speed, we transformed them using a trigonometric function into WY (i.e., wind speed in north–south (N–S) direction) and WX (i.e., wind speed in east–west (E–W) direction), where WY is the product of wind speed and the cosine of wind direction, and WX the multiplication of wind speed by the sine value of wind direction.

**Figure 6:**
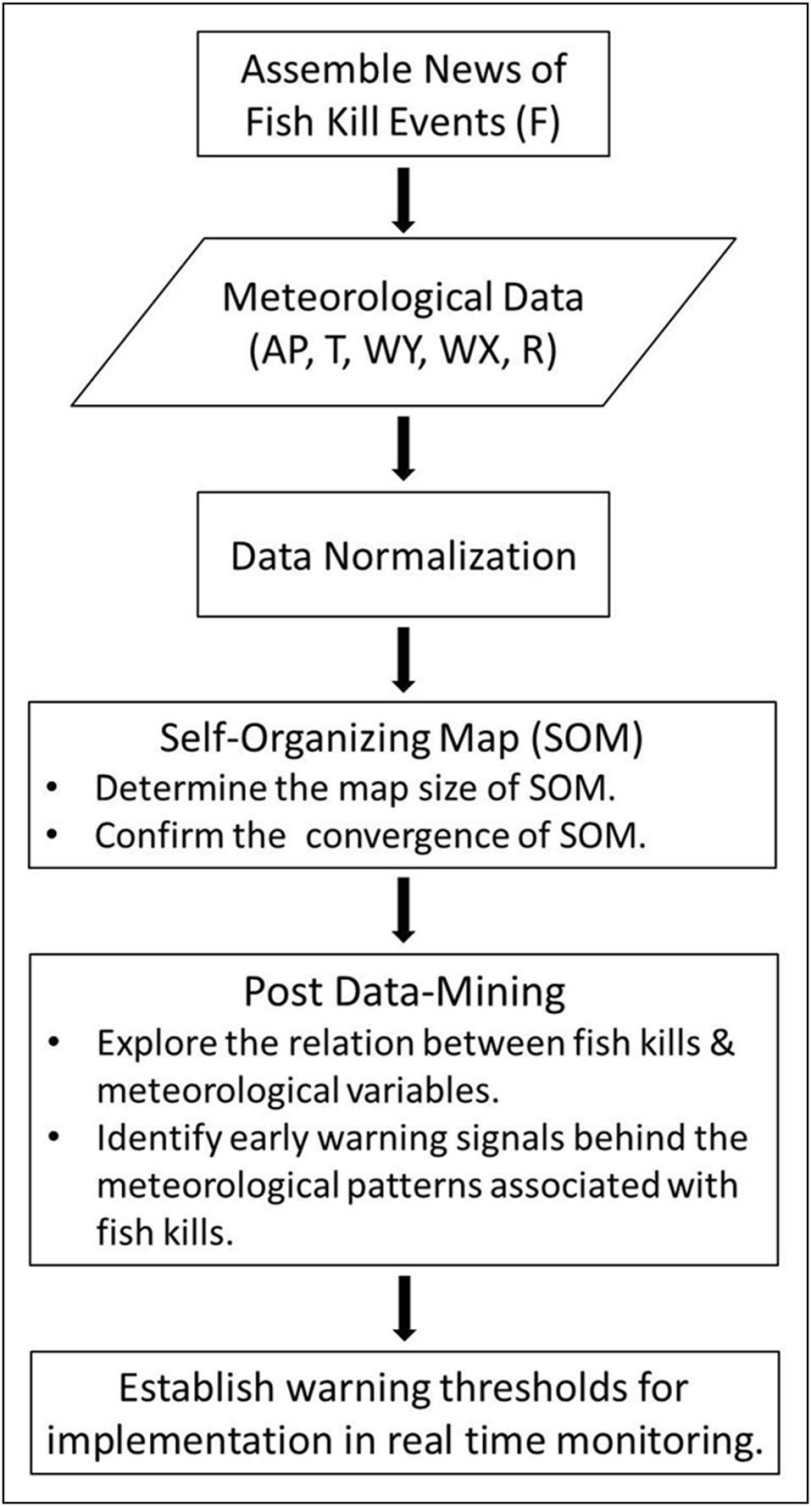
Flowchart of this study.

Each variable was normalized to the range of 0 to 1, preventing a biased interpretation in the formation of data analysis^49,53^. The normalized data of the implicit hourly meteorological variables (i.e., AP, T, WY, WX, and R) were paired with the normalized F to implement the SOM using MATLAB R2015b software. We applied an unsupervised competitive learning algorithm for clustering the nonlinear interrelationship into a hexagonal lattice topological map using a Gaussian neighborhood function. Then based on the SOM clustering results, we returned to the original data and performed a data-mining task to investigate the linkage between fish kill occurrence to meteorological factors of air pressure, temperature, wind speed, wind direction, and precipitation. Based on the clustered fish kill types by SOM, we performed a risk analysis comparing meteorological patterns behind fish kills to their normal conditions using t-test. Lastly, we established warning thresholds by applying values of positive or negative one standard deviation of the normal non-kill conditions as early warning signals for timely preventative actions (Fig. 6).

## Data Availability

The data that support the findings of this study are available from the Open Weather Data of Taiwan (https://opendata.cwb.gov.tw/dataset/climate?page=1). Restrictions may apply to the availability of these data with the permission of the Open Weather Data of Taiwan.
